# T-G-A Deficiency Pattern in Protein-Coding Genes and Its Potential Reason

**DOI:** 10.3389/fmicb.2022.847325

**Published:** 2022-05-04

**Authors:** Yan-Ting Jin, Dong-Kai Pu, Hai-Xia Guo, Zixin Deng, Ling-Ling Chen, Feng-Biao Guo

**Affiliations:** ^1^School of Life Science and Technology, University of Electronic Science and Technology of China, Chengdu, China; ^2^Department of Respiratory and Critical Care Medicine, Zhongnan Hospital of Wuhan University, Key Laboratory of Combinatorial Biosynthesis and Drug Discovery, Ministry of Education and School of Pharmaceutical Sciences, Wuhan University, Wuhan, China; ^3^Agricultural Bioinformatics Key Laboratory of Hubei Province, College of Informatics, Huazhong Agricultural University, Wuhan, China

**Keywords:** T-G-A deficiency, stop codons, premature protein, termination, codon position

## Abstract

If a stop codon appears within one gene, then its translation will be terminated earlier than expected. False folding of premature protein will be adverse to the host; hence, all functional genes would tend to avoid the intragenic stop codons. Therefore, we hypothesize that there will be less frequency of nucleotides corresponding to stop codons at each codon position of genes. Here, we validate this inference by investigating the nucleotide frequency at a large scale and results from 19,911 prokaryote genomes revealed that nucleotides coinciding with stop codons indeed have the lowest frequency in most genomes. Interestingly, genes with three types of stop codons all tend to follow a T-G-A deficiency pattern, suggesting that the property of avoiding intragenic termination pressure is the same and the major stop codon TGA plays a dominant role in this effect. Finally, a positive correlation between the TGA deficiency extent and the base length was observed in start-experimentally verified genes of *Escherichia coli* (*E*. *coli*). This strengthens the proof of our hypothesis. The T-G-A deficiency pattern observed would help to understand the evolution of codon usage tactics in extant organisms.

## Introduction

In the early 1980s, Grantham and colleagues (Grantham et al., 1980a,b) proposed that the genome rather than the individual gene is the unit of codon usage selection and each genome seems to have its general pattern of codon usage. Soon after, it was exposited that there is also a sub strategy (or pattern) within a genome that the choice of the degenerate (third) position strongly correlates with the expression level of the gene (Grantham et al., 1981; Ikemura, 1981a). Highly expressed genes, particularly encoding ribosomal proteins, choose their synonymous codons based on the corresponding tRNA level. These observations led to a well-accepted theory: translation efficiency exerts selection on synonymous codon usage within a genome (Chen et al., 2017) and hence highly expressed genes adapt their degenerate position to the major tRNA (Ikemura, 1981b; Hanson and Coller, 2018). Based on this proposal, optimizing synonymous codon has been widely used as a method to increase the expression level of the target genes in bioengineering (Zalucki et al., 2011; Yang and Zhang, 2017). Latest studies revealed that the general codon usage pattern of a certain genome is determined by its G+C content (Zhou et al., 2014; Romiguier and Roux, 2017). It was also found that the asymmetric replication mechanism would cause genes on the leading strands to contain more G than C and T than A, particularly at the third codon positions; and the case is opposite for the lagging strand genes (Mrazek and Karlin, 1998). This phenomenon of strand composition bias is often explained as the consequence of varied mutation rates (Frank and Lobry, 1999; Zhao et al., 2015). However, recent research successfully revealed the major role of minimizing energy costs on this property (Chen et al., 2016).

Are there other factors that could ubiquitously influence the codon usage of genes? In this work, we focus on the translation terminus mechanism and aim to check whether it brings pressure on codon usage. The last codon of every gene sequence signals the translation terminus. When meeting this codon, the ribosomal subunits will disassociate and release the amino acid chain. The stop codon is TGA, TAA, or TAG. If there appear one or more mutations, which generate an additional stop codon along with the correct phase before the actual terminus of a gene, its translation would end at this site (Si et al., 2016). In most cases, the protein of this gene will fail to normally fold and hence could not perform its natural function (Kim et al., 2015). These events are called premature proteins (Stalder and Mühlemann, 2008) and they are adverse to the host (Lueck et al., 2019; Abrahams et al., 2021; Den Hoed et al., 2021; Supek et al., 2021; Szpak et al., 2021). We think that codon usage should form in one order to avoid the appearance of additional stop codons in the same frame of the terminus stop codon.

If there indeed exists pressure of avoiding pre-termination of translation, we speculate that it will generate the following two results: (i) if a codon in a gene has a low frequency, then all of its three individual nucleotides would also have low frequencies. Gene requests the elimination of in-frame stop codons within coding regions, and hence there will be less frequencies of nucleotides matching the stop codons at all three codon positions. In detail, at the first codon position, nucleotide T (the first nucleotide for all the three stop codons) will be much less than C, G, and A. If this is not really 100%, at least T will be less than A. Similarly, there will be less G and/or A than the other nucleotides at the other two codon positions. (ii) If a gene is longer, there will be more stop codons appearing in its sequences with random nucleotide distribution. Hence, the stop codon corresponding nucleotides will have lower frequencies than that of short genes. Here, we test our two speculations with 19,911 prokaryotic genomes and a gene set with experimentally validated 5′ terminals of *Escherichia coli* (*E. coli*). Our result illustrates that the translation terminus pressure significantly influences codon usage of genes.

## Materials and Methods

### Bioinformatics Data Source

We extracted genomic sequences and annotated gene coordinates contained in (^*^.gbff) files of bacteria and archaea from the GenBank database in March 2018 (Benson et al., 2018; http://guolab.whu.edu.cn/genome/listbateria.html). There are a total of 32,319 files, and among them, 19,911 genomes have complete sequences and gene annotation information. Therefore, nucleotide frequency analyses were based on 19,911 prokaryotic genomes. The accession numbers and nucleotide frequency information were listed in Supplementary Table 1. To ensure the analyses are more reliable, we extract the genes with identified products (i.e., analyses were only restricted to function-known genes) and remove the genes whose lengths are not multiples of three or with abnormal terminators to compose our data set. In addition, we use the EcoGene database as a reliable data set of the translation start in *E. coli* (Zhou and Rudd, 2013), which was maintained to continuously improve the structural and functional annotation of *E. coli* K-12 MG1655. This dataset contains 513 proteins with experimentally determined N-terminus.

### Statistical Analysis

The Wilcoxon Signed-Rank Test was used to check the statistical significance of nucleotide frequency difference between the two groups of genes or genomes. The Pearson correlation coefficient was used to evaluate the correlation strength between two variables.

## Results

### Most Genomes Were Found to Have the Least Nucleotides Corresponding to T-G-A

**Figure 1:**
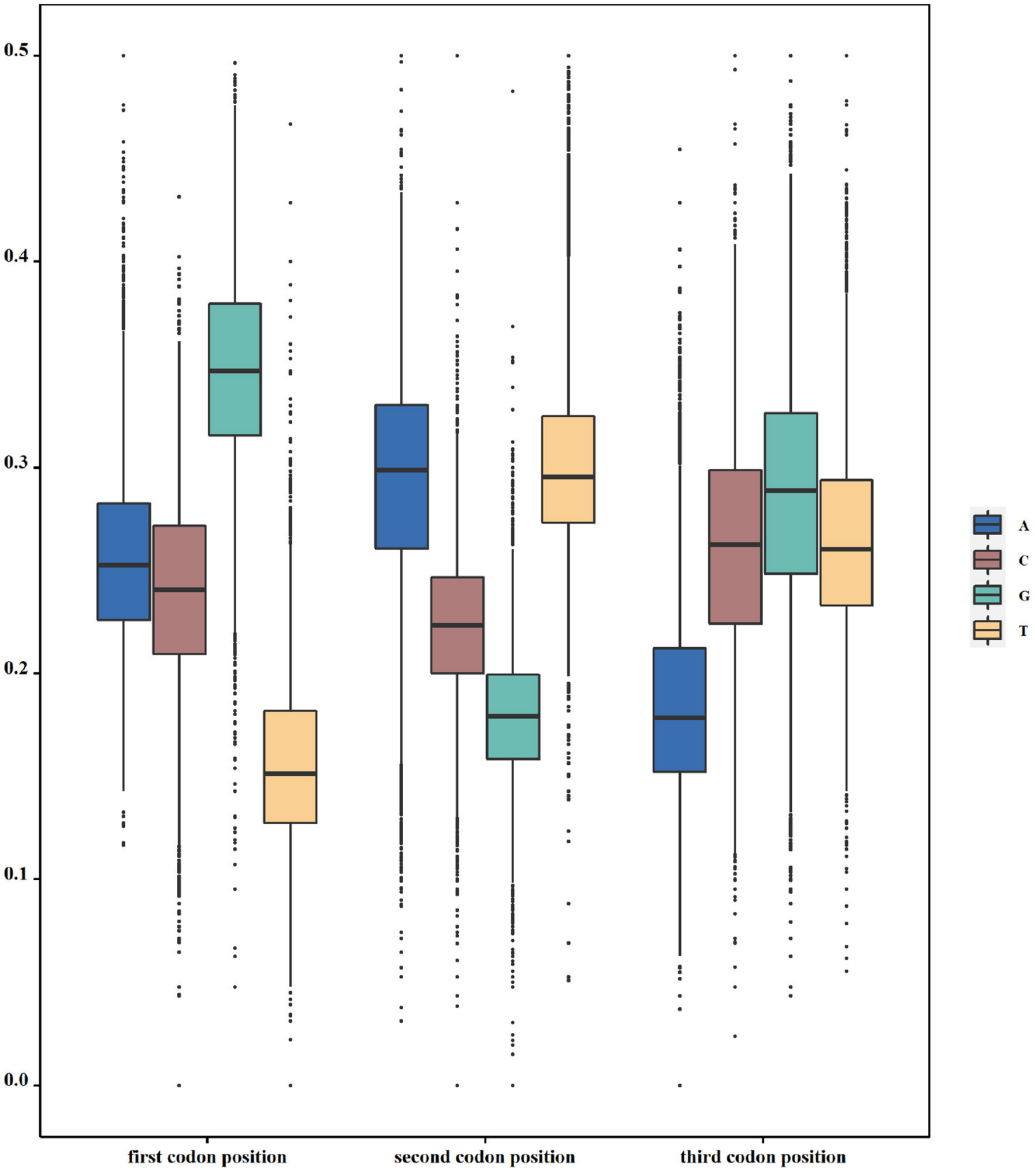
Usage of nucleotides in triplet codons. The y axis denotes the frequency of one specific nucleotide at one specific codon position. In *Escherichia coli* (*E. coli*), T, G, and A are used the least at the first, second, and third positions of triple codons, respectively.

According to our hypothesis, if there indeed exists a selection pressure for avoiding translation terminal during the inner region of coding genes, they will have the least frequencies of nucleotide corresponding to the stop codon at each codon position. To validate this conjecture, we calculated the frequencies of all four nucleotides at three codon positions in each genome. That is to say, we take a genome as the calculating unit and sum up all individual nucleotides in all its function-known genes. As Table 1 shows, T constitutes the least nucleotides at the first codon position in 69.7% of 19,911 genomes, whereas the other three nucleotides, as a whole, constitute the least ones only in 30.3% of genomes. At the second codon position, 99.1% of genomes have the least nucleotide of G and 52.7% of genomes have the least nucleotide of A at the third codon position. Differences in T-G-A with other nucleotides at the corresponding site of triple codons are statistically significant (*p* < s0.05). As an example in *E. coli*, the frequency distribution of all four nucleotides at all codon positions is shown in Figure 1. Based on these results, it is reasonable to conclude that our first speculation is basically validated.

**Table 1:** Number and percentage of bacterial genomes with each nucleotide showing the least at each codon position^a^.

	**A**		**T**		**C**		**G**	
	**Genome** **number**	**Genome** **percentage**	**Genome** **number**	**Genome** **percentage**	**Genome** **number**	**Genome** **percentage**	**Genome** **number**	**Genome** **percentage**
First^b^	0	0	**13,872**	**69.7**	6,039	30.3	0	0
Second	176	0.9	0	0	3	0.0	**19,732**	**99.1**
Third	**10,496**	**52.7**	827	4.2	6,224	31.3	2,364	11.9

a *For each genome, we calculated frequency of four nucleotides at each codon position and chose the least nucleotides for this genome*.

b *Taking the first codon position as an example, we explain the meaning of the genome number and genome percentage. As we know, a total of 19,911 genomes are involved. Among them, 13,872 genomes have T as the least nucleotide out of the four nucleotides at the first codon position and 6,039 genomes have C as the least nucleotide at this codon position. No genomes have A and G as the least nucleotide at this position. In other words, T constitutes the least nucleotides at the codon position in more genomes than A, C, and G*.

*The bold values indicate the least nucleotides at each codon position*.

However, there are some outliers deviating from our rule. For example, a fraction of genomes still has the least nucleotide of C at the first codon position. We consider that the appearance of these exceptional genomes may be caused by their GC content and accordingly found that 6,039 non-T (do not have the least nucleotide of T) genomes have an average GC content of 37.8%, which is 20% less (*t*-test, *p*-value = 0) than the retaining genomes. Almost all of these exceptional genomes are AT-richer (minimum AT content is 49.2% among them) and A plus T constitute the major nucleotides, hence nucleotide composition pressure will be very difficult to make the T being the least one. In these cases, we loosen the restriction and only require T to have lesser frequencies than A at the first codon positions. With this adaption, all of the 19,911 genomes with no exceptions satisfy the rule that has the least T or less T than the counterpart nucleotide A at the first codon position. Similar events are observed when studying the second and third codon positions. The frequency of second position's G is the least in 99.1% of genomes and less than C in almost all genomes (99.97%). The frequency of the third position's A is the least or less than T in 85.9% of genomes.

### Check Genes Without TGA as the Terminators and Confirm the Uniformed Property of Genes With Different Stop Codon Types

As mentioned above, most genomes tend to avoid the T-G-A nucleotide at each codon position and those outliers at least have T less than A, G less than C, and A less than T. It seems that there is a uniform pressure from avoiding the TGA similar codon. However, we know that the three codons (TGA, TAA, and TAG) could act as terminators and the actual terminator for each gene relies on a specific gene sequence. According to our statistics, among the 40,648,966 genes in 19,911 genomes, TGA acts as terminators in 45.7% of the total genes and TAA and TAG play roles in 37.0 and 17.3% of the total genes. Then, we wonder whether each gene has nucleotides to coincide with its specific terminator type or all genes suffer pressure with the same property. Hence, we classify all genes into three types according to their factual stop codons and calculate their nucleotide frequencies for each gene and sum them up in each group. As Table 2 shows, all groups of genes tend to use the least G at the second codon position and A at the third codon position. Even in the TAA-ending and TAG-ending groups, for the second codon position G, the least genes hold a higher ratio than that in the TGA-ending groups. Hence, it means TAA-ending and TAG-ending genes would not be requested to have the nucleotide frequency as T-A-A or T-A-G deficiency instead of T-G-A deficiency. Therefore, it is reasonable to conclude that the property of avoiding an intragenic termination pressure is the same and it does not depend on the genes' specific stop codon type.

**Table 2:** Percentage of genes with nucleotides with the least frequency at the first, second, and third positions in three groups of genes^a^.

	**First position**		**Second position**		**Third position**	
	**Least nucleotide**	**Gene percentage**	**Least nucleotide**	**Gene percentage**	**Least nucleotide**	**Gene percentage**
TAG group	T	0.728	G	0.730	A	0.551
TGA group	T	0.845	G	0.627	A	0.661
TAA group	T	0.620	G	0.804	A	0.380

a *To save space and improve the readability, only the nucleotide with the highest percentage of genes was shown*.

### Gene Length Regulates T-G-A Frequency Furthermore

**Figure 2:**
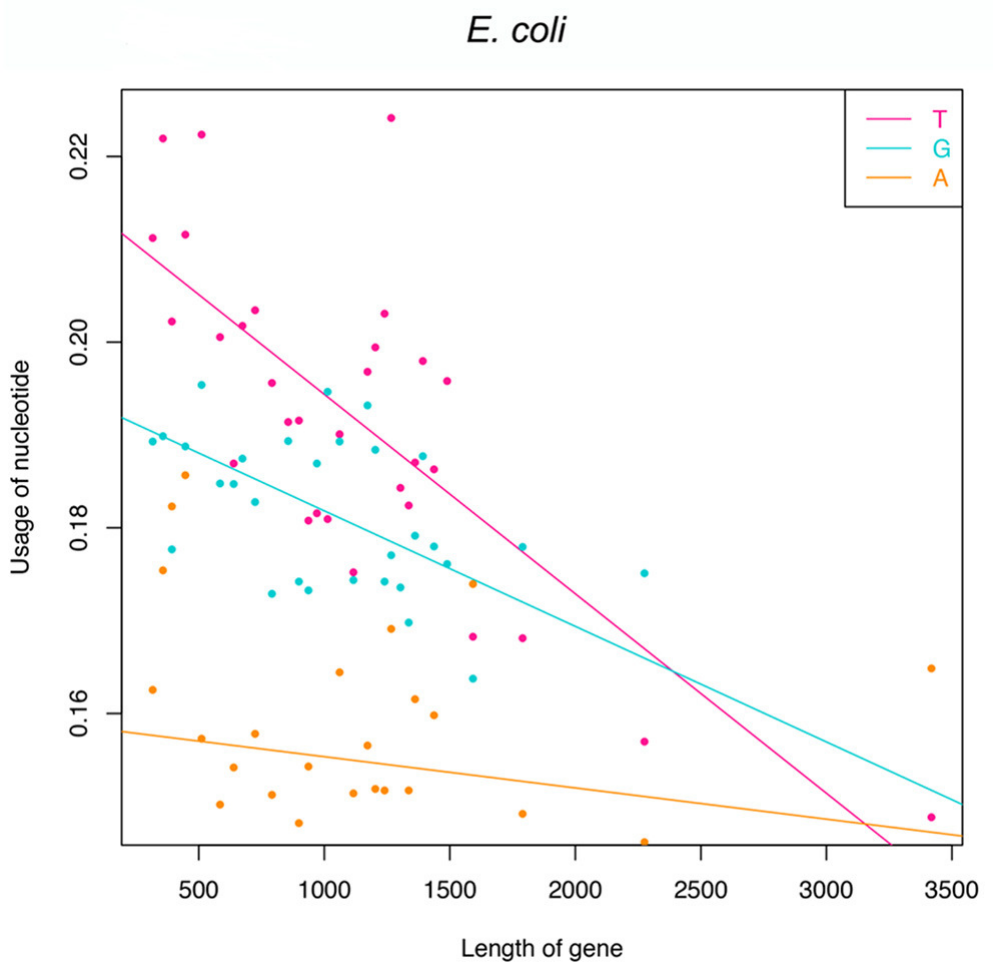
Linear regression analyses of frequencies of T, G, and A against gene length. Note that 513 genes are divided into 30 groups based on their lengths.

Functional genes indeed have fewer T-G-A frequencies at the first to the third codon position because of the pressure to avoid premature termination. However, it should be researched whether there are some regulatory factors that will cause different absence levels among genes? In detail, will longer genes suffer more strict pressure and have much fewer frequencies of T-G-A? If longer genes have a similar T-G-A frequency to shorter genes, statistically, they will generate more than one stop codons. To check this point, we resort to translation-start verified genes of *E. coli* since many genes in the genome database would have an inaccurate annotation of start sites and their accurate lengths would be affected. The Pearson correlation was used to capture the trend of T-G-A frequencies varying with the increasing length of genes. To decrease the noise and eliminate the burrs, all 513 genes are sorted by length and divided into 30 groups (each group contains 17 genes, and the last 3 genes are added to the 30th group), and the mean nucleotide frequencies and gene length are calculated for each group. The results of Pearson correlation show that the frequencies of T (*r* = –0.64, *p* = 4.24e−7), G (*r* = –0.44, *p* = 1.17e−3), and A (*r* = –0.33, *p* = 1.68e−2; Figure 2) are negatively correlated with gene length, particularly at the first two codon positions. In other words, longer genes have much fewer frequencies of T-G-A at the first to the third codon position than shorter genes.

We also investigate the nucleotide frequency changes against the distance to the factual stop codons. In this analysis, we divide each gene into 15 sections according to the order from the left to the right. Then, we calculated the frequency of nucleotide T at the first codon position, G at the second codon position, and A at the third codon position. Note that we used the average values of these frequencies of the 513 genes. This time, we do not observe any clear variation besides a slight frequency increment at the right terminal (Supplementary Figure 1).

## Discussion

A protein will be cut short if a stop codon appears in the inner coding region (Si et al., 2016). Premature translation termination will decrease the fitness and even be lethal for two reasons: (1) translating useless proteins wastes energy and is detrimental to the growth and reproduction of organisms; (2) the truncated proteins might interact with other proteins or genes and might influence the fitness or even contribute to the death of organisms (Stalder and Mühlemann, 2008). Thus, we presume that functional genes should try to avoid inner stop codons and longer genes will have a lower possibility of nucleotides matching to terminators than shorter genes. In this work, we successfully validated our hypothesis by nucleotide frequency analyses in 19,911 prokaryotic genomes. Furthermore, we reveal that genes with different stop codons have the same least nucleotide at the latter two codon positions. Therefore, this type of selection evolved the same property and it just conforms to the major terminator TGA and does not depend on a specific stop codon. It should be noted that in higher-eukaryotic humans, we also observe a similar T-G-A deficiency pattern (Supplementary Table 2).

The Pearson correlation analyses revealed that longer genes have stricter pressure than shorter genes. This result also verifies that the T-G-A pattern is indeed caused by the pressure of avoiding premature. Many researchers have revealed that both highly expressed genes and essential genes have median or relatively small lengths compared to other genes (Gong et al., 2008; Grishkevich and Yanai, 2014). Therefore, this study observed the severer T-G-A deficiency of longer genes could not be attributed to any functional selection.

Codon usage in a genome or a gene is determined by many factors (Chaney and Clark, 2015; Ling et al., 2015; Novoa et al., 2019) including the major one, GC content (Ho and Hurst, 2021). T-G-A deficiency, as a general pattern, would be determined by the pressure of avoiding premature proteins. However, we do not disregard the effects of other mutational or functional factors on codon usage. We deemed that codon usage of a gene or a genome should reflect the equilibrium consequent of complex factors, and here we revealed one previously not observed or not explicitly concluded factor. As we can see, A at the third codon position does not hold such a strong association with the gene length as the first two codon positions, and this may be due to the degenerate site suffering from a selection of translation efficiency and tending to use synonymous nucleotides coinciding with the major tRNA (Ikemura, 1981b). This type of translation selection would compromise the influence of avoiding premature. We hope here that the observed T-G-A-deficiency pattern would help to understand evolved codon usage tactics of extant organisms.

## Data Availability Statement

The original contributions presented in the study are included in the article/Supplementary Material, further inquiries can be directed to the corresponding author/s.

## Author Contributions

F-BG designed and coordinated this project. Y-TJ did the computation work. D-KP and H-XG double checked the results. F-BG, Y-TJ, and D-KP analyzed the results and drafted the manuscript. L-LC and F-BG revised the manuscript with comments from other authors. All authors contributed to the article and approved the submitted version.

## Funding

This work was supported by the Natural Science Foundation of China [31871335].

## Conflict of Interest

The authors declare that the research was conducted in the absence of any commercial or financial relationships that could be construed as a potential conflict of interest.

## Publisher's Note

All claims expressed in this article are solely those of the authors and do not necessarily represent those of their affiliated organizations, or those of the publisher, the editors and the reviewers. Any product that may be evaluated in this article, or claim that may be made by its manufacturer, is not guaranteed or endorsed by the publisher.

## Supplementary Material

Supplementary Table 1 Accession numbers species name, genomic GC content, no. of analyzed genes, the average frequency of each stop codon and nucleotide frequency information of 19,911 prokaryotic genomes.

Supplementary Table 2 Percentage of human genes with nucleotides used least at the first, second, and third position in three groups of genes.

Supplementary Figure 1 Nucleotide frequency changes against the distance to the factual stop codons. Note that number 1 denotes the most left fragment and 15 as the most right fragment.
