# Supplementary material for: T-G-A Deficiency Pattern in Protein-Coding Genes and Its Potential Reason
Source: Front Microbiol. 2022 May 4;13:847325. doi: 10.3389/fmicb.2022.847325 (PMC9116502; doi:10.3389/fmicb.2022.847325)
Supplement: Supplementary Table 2 — Percentage of human genes with nucleotides used least at the first, second, and third position in three groups of genes. [file Table_2.docx]

Table S2. Percentage of human genes with nucleotides used least at the first, second and third position in three groups of genes.

|  | **First position** | | **Second position** | | **Third position** | |
| --- | --- | --- | --- | --- | --- | --- |
|  | Least nucleotide | Gene percentage | Least nucleotide | Gene percentage | Least nucleotide | Gene percentage |
| **TAG group** | T | 0.801 | G | 0.678 | A | 0.574 |
| **TGA group** | T | 0.793 | G | 0.620 | A | 0.578 |
| **TAA group** | T | 0.733 | G | 0.711 | A | 0.402 |
