# Supplementary figures and images for: T-G-A Deficiency Pattern in Protein-Coding Genes and Its Potential Reason
Source: Front Microbiol. 2022 May 4;13:847325. doi: 10.3389/fmicb.2022.847325 (PMC9116502; doi:10.3389/fmicb.2022.847325)

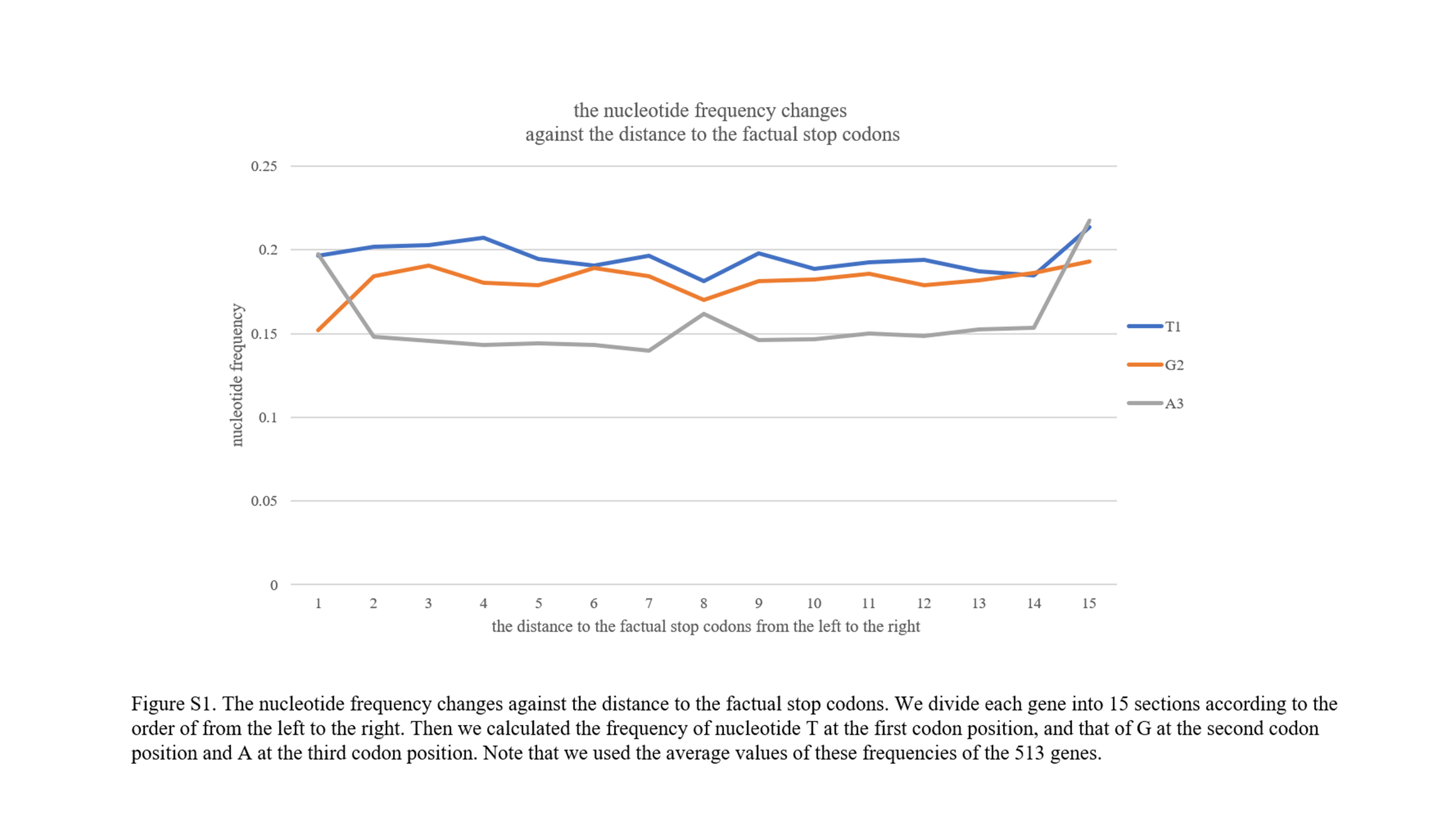

Supplement: Supplementary Figure 1 — Nucleotide frequency changes against the distance to the factual stop codons. Note that number 1 denotes the most left fragment and 15 as the most right fragment. [file Image_1.png]
